# Supplementary material for: Conditional chemoconnectomics (cCCTomics) as a strategy for efficient and conditional targeting of chemical transmission
Source: eLife. 2024 Apr 30;12:RP91927. doi: 10.7554/eLife.91927 (PMC11060718; doi:10.7554/eLife.91927)
Supplement: Figure 3—source data 3. [file elife-91927-fig3-data3.zip › Figure 3 source data3 part 1.zip!/Figure 3-Source data 3-part1/1082-9-CRISPResso_Report_zod2ewbs.zip!/CRISPResso_on_1082_10/2b.Nucleotide_percentage_quilt_around_sgRNA_catccaggcagatccaggtg.pdf]

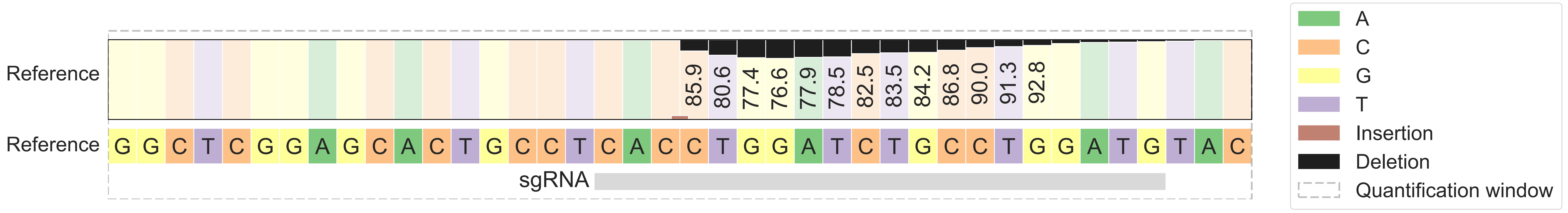
Reference
Reference
G G C T C G G A G C A C T G C C T C A C C T G G A T C T G C C T G G A T G T A C
85.9 80.6 77.4 76.6 77.9 78.5 82.5 83.5 84.2 86.8 90.0 91.3 92.8
sgRNA
A
C
G
T
Insertion
Deletion
Quantification window
